# The coMforT study of a trauma-informed mindfulness intervention for women who have experienced domestic violence and abuse: a protocol for an intervention refinement and individually randomized parallel feasibility trial

**DOI:** 10.1186/s40814-019-0527-1

**Published:** 2020-02-28

**Authors:** Katherine Pitt, Gene S Feder, Alison Gregory, Claire Hawcroft, David Kessler, Alice Malpass, Sarah Millband, Richard Morris, Stan Zammit, Natalia V Lewis

**Affiliations:** 1 Centre for Academic Primary Care, Bristol Medical School (Population Health Sciences), University of Bristol, Canynge Hall, 39 Whatley Road, Bristol, BS8 2PS UK; 2 National Institute for Health Research Bristol Biomedical Research Centre, University Hospitals Bristol NHS Foundation Trust and University of Bristol, Bristol, UK; 3 Division of Psychological Medicine and Clinical Neurosciences, Cardiff University, Cardiff, UK

**Keywords:** Domestic violence and abuse (DVA), Trauma, Post-traumatic stress disorder (PTSD), Mindfulness, Mindfulness-based cognitive therapy (MBCT), Intervention development, Feasibility trial

## Abstract

**Background:**

Domestic violence and abuse (DVA) is common and destructive to health. Post-traumatic stress disorder (PTSD) is a major mental health consequence of DVA. People who have experienced DVA have specific needs, arising from the repeated and complex nature of the trauma. The National Institute for Health and Care Excellence recommends more research on the effectiveness of psychological interventions for people who have experienced DVA. There is growing evidence that mindfulness-based interventions may help trauma symptoms.

**Methods:**

Intervention refinement and randomized controlled feasibility trial. A prototype trauma-informed mindfulness-based cognitive therapy (TI-MBCT) intervention will be co-produced following qualitative interviews and consensus exercise with stakeholders. Participants in the feasibility trial will be recruited from DVA agencies in two geographical regions and randomized to receive either TI-MBCT or usual care (self-referral to the Improving Access to Psychological Therapies (IAPT) service). TI-MBCT will be delivered as a group-based eight-week program. It will not be possible to blind the participants or the assessors to the study allocation. The following factors will inform the feasibility of progressing to a fully powered trial: recruitment, retention, intervention fidelity, and the acceptability of the intervention and trial design to participants. We will also test the feasibility of measuring the following participant outcomes before and 6 months post-randomization: PTSD, dissociative symptoms, depression, anxiety, DVA re-victimization, self-compassion, and mother-reported child health. Process evaluation and economic analysis will be embedded within the feasibility trial.

**Discussion:**

This study will lead to the development of a TI-MBCT intervention for DVA survivors with PTSD and inform the feasibility and design of a fully powered randomized controlled trial (RCT). The full trial will aim to determine the effectiveness and cost-effectiveness of a TI-MBCT intervention in improving the clinically important symptoms of PTSD in DVA survivors.

**Trial registration:**

ISRCTN, ISRCTN64458065, Registered 11 January 2019.

## Background

### Mental health consequences of domestic violence and abuse

Domestic violence and abuse (DVA) is a major public health and clinical problem which is experienced by 1 in 4 women and 1 in 7 men in England and Wales [1]. Although DVA is experienced by both women and men, the impact on health is greater among women [2]. Between 6% and 17% of women attending primary care report having been abused by a partner in the past year [3]. The single biggest cost associated with DVA is to the NHS: £1.7 billion per year, with the major cost borne by acute trusts and primary care. Mental health service costs are estimated to be an additional £176 million [4].

A major mental health consequence of DVA experienced by women is post-traumatic stress disorder (PTSD), with a 7.3 odds ratio for the experience of intimate partner violence in women with PTSD [5]. Another study found a two-fold increase in the prevalence of PTSD among women with higher levels of exposure to DVA [6]. A psychiatric morbidity survey in England found that 12.6% of women aged 16 to 24 screened positive for PTSD [7]. Symptoms of PTSD include reliving the traumatic experience, hypervigilance, avoidance, and cognitive distortions often related to beliefs about safety, power, and self-worth that impair the ability to break the cycle of abuse [8, 9]. Although research findings suggest a spontaneous recovery of PTSD in some people who have experienced DVA, a significant number (46.8%) of women exhibit chronic PTSD [10].

In contrast to single-event trauma, DVA occurs repeatedly and often increases in severity over time [11]. Women who have experienced DVA represent a distinct patient group due to the complexity of their trauma and its specific impact on affect regulation, changes in consciousness, sense of self, relationships, and belief systems [12]. Survivors of DVA often have numerous and cumulative psychosocial stressors, including poverty, lack of social support, parenting stress, and ongoing contact with the perpetrator through child contact [9, 13]. For these women, accessing standard trauma-focused treatments can be practically difficult and emotionally problematic [14–16]. There is also the need to address safety and the risk of re-traumatization if there is ongoing contact with the perpetrator [17]. The stigma associated with mental health symptoms also leads to decreased use of mental health services; women who have experienced DVA may fear that a mental health diagnosis will result in their children being removed from their care [13, 18].

### Impact on children

Children in families affected by DVA are at higher risk of developmental and behavioral problems, compromising their lifelong wellbeing and functioning [19]. Children whose mothers have experienced DVA and have PTSD and depression are at higher risk of behavior problems [20].

### Evidence-based interventions for PTSD

The National Institute for Health and Care Excellence (NICE) guidance on PTSD advocates individual trauma-focused cognitive behavior therapy (TF-CBT) and eye movement desensitization and reprocessing (EMDR) as evidence-based treatment options [21]. A Cochrane review [22] showed that both therapies performed better than usual care for reducing symptoms of PTSD. Both therapies use exposure work, requiring participants to directly confront their traumatic stories. In addition, many of the study populations identified in the review had experienced single event traumas. A significant proportion of PTSD sufferers either do not seek help, drop out of or decline these treatments, or continue to meet diagnostic criteria for PTSD after trauma-focused interventions [23]. The NICE guidance recommends more research on the effectiveness of psychological interventions modified for DVA [24].

In contrast to trauma-focused approaches, mindfulness-based interventions do not include exposure work and therefore could be more acceptable to survivors of DVA. Mindfulness-based interventions have recently received increased attention for helping patients who have experienced trauma [25, 26]. A recent systematic review [25] which included four studies with people who have experienced childhood abuse and DVA found some evidence that mindfulness-oriented interventions may decrease PTSD symptoms, although most studies were underpowered and had methodological weaknesses. The review identified the need for further modification of mindfulness interventions for PTSD and further adequately powered randomized controlled trials (RCTs) of the modified interventions.

### Pre-protocol development of a mindfulness intervention for DVA trauma

This study is built on pre-protocol development work carried out in 2014–2015: (i) a synthesis of prior literature on mindfulness-oriented interventions for DVA and PTSD, (ii) consultations with a group of DVA survivors on potential adaptations to standard mindfulness treatment, (iii) consultation with mental health professionals on the rationale behind mindfulness approaches for PTSD in the DVA population, (iv) consultation with DVA advocates on the safety of mindfulness approaches for women who have experienced DVA, and the feasibility of recruitment through third sector DVA agencies. Informed by this work, a mindfulness teacher with expertise and experience in trauma developed a trauma-informed intervention by modifying the standard manual for preventing depression relapse (mindfulness-based cognitive therapy (MBCT)) (Millband S.: How can an adapted MBCT course meet the specific vulnerabilities of women survivors of domestic violence and abuse? Unpublished thesis: Bangor University; 2015). This adaptation process was overseen by a mindfulness-based supervisor with expertise in trauma. We call this modification a trauma-informed mindfulness-based cognitive therapy (TI-MBCT) prototype 1. This prototype intervention was pre-piloted with a group of women who had experienced DVA and their feedback was obtained through a group interview. The feedback from the pre-pilot testing suggested that the prototype TI-MBCT program may be acceptable to a DVA population and might be effective but required robust evaluation with a randomized controlled design. Our pre-protocol development work identified several areas of uncertainty about the future trial associated with:

1. Choice of the control intervention. In a definitive trial, the control intervention will be an evidence-based manualized psychological intervention for PTSD currently available within NHS Improving Access to Psychological Therapies (IAPT) services. Our primary intention was to use trauma-informed cognitive behavioral therapy (TI-CBT). However, consultations with service user and professional stakeholders revealed that in DVA agencies women with mental health problems are signposted to their GPs and IAPT services. The latter offers varied treatments (e.g., trauma counseling, TF-CBT, EMDR) depending on an individual’s symptoms and needs and practitioners’ availability.
2. Recruitment of DVA survivors with PTSD through third sector DVA service
3. The randomization of DVA survivors with PTSD
4. Retention of DVA survivors with PTSD in the trial
5. Uptake, retention, and acceptability of the modified MBCT intervention to DVA survivors with PTSD
6. Data collection methods.

The coMforT (Mindfulness for Trauma) study addresses these areas of uncertainty through further intervention development work with stakeholders followed by feasibility testing of the refined intervention.

## Methods/design

### Study aim/objectives

The coMforT study aims to (i) produce a TI-MBCT program that is acceptable to DVA survivors with PTSD and feasible to deliver and (ii) establish the feasibility of a definitive trial of a TI-MBCT intervention vs control intervention for this population.

The objectives of the intervention development work are to

1. Elicit service users’ and professional stakeholders’ suggestions for recruitment and retention of DVA survivors with PTSD and their uptake and engagement with the prototype TI-MBCT
2. Elicit service users’ and professional stakeholders’ suggestions for additional refinements of the prototype TI-MBCT that will increase uptake and engagement with the intervention.
3. Produce TI-MBCT manual for feasibility testing.

The objectives of the subsequent feasibility trial are to

1. Determine which psychological intervention currently offered within the NHS IAPT service can be used as control intervention in a definitive trial, and explore the practicality of referring study participants to this control intervention
2. Assess the feasibility of recruiting participants through third sector organizations providing health and social care to DVA victims and survivors
3. Evaluate the acceptability of randomization to participants
4. Determine if it is possible to retain DVA survivors with PTSD to follow-up
5. Refine the orientation process for the TI-MBCT intervention
6. Examine if it is possible to enroll and retain DVA survivors with PTSD in TI-MBCT groups
7. Evaluate the acceptability of the TI-MBCT intervention to DVA survivors with PTSD
8. Determine the practicalities of delivering TI-MBCT in the community setting
9. Produce TI-MBCT manual for the full-size trial
10. Examine the acceptability and completeness of the data collection methods for a definitive trial
11. Estimate variance and distribution of quantitative outcomes in the feasibility trial data to inform the design and sample size calculation in the future definitive trial.

A traffic light system will be used to review progression criteria to a full trial, whereby “green” indicates that it is feasible to conduct a definitive trial with the current trial design and procedures, “amber” indicates that improvements are required before conducting a definitive trial, and ‘red’ indicates that it is not feasible to progress to a definitive trial (see Table 1). The independent study steering group will be consulted about whether progressing to a definitive trial is justified.

**Table 1:** Criteria for progression from feasibility trial to full-size trial

Progression criteria	Measurement	Green	Amber	Red
Recruitment	Number of participants recruited over 6 months	24	12–23	< 12
Recruitment	Qualitative process evaluation	Most participants find the recruitment procedures acceptable or only minor amendments needed.	Participants’ views on acceptability conflicting or larger changes needed.	Most participants find unacceptable or changes needed unfeasible.
Randomization	Qualitative process evaluation	Most participants understand the randomization process and find it acceptable.	Participants’ understanding and views on acceptability conflicting.	Most participants do not understand process or find it unacceptable
Follow-up (total and by trial arms)	Proportion of enrolled participants providing primary outcome data at 6 months post-randomization	> 50%	31–50%	≤ 30%
Follow-up (total and by trial arms)	Qualitative process evaluation	Most participants find the follow up process acceptable or only minor amendments needed.	Participants’ views on acceptability conflicting or larger changes needed.	Most participants find processes unacceptable or changes needed unfeasible.
Uptake of TI-MBCT	Proportion of participants who took up the mindfulness group out of those randomized in the intervention arm [27, 28]	> 70%	50–69%	< 50%
Retention in TI-MBCT group	Proportion of participants in the intervention arm who received at least four sessions [29, 30]	≥ 60%	40–59%	< 40%
TI-MBCT acceptability	Qualitative process evaluation	Most participants find the mindfulness group acceptable or only minor amendments needed.	Views on acceptability conflicting or larger changes needed.	Most participants find unacceptable or changes needed unfeasible.
Data collection methods	Qualitative process evaluation	Most participants find the data collection procedures acceptable or only minor amendments needed.	Views on acceptability conflicting or larger changes needed.	Most participants find unacceptable or changes needed unfeasible.

Note: *TI-MBCT* trauma-informed mindfulness-based cognitive therapy

### Study design

The study consists of two components: (1) refining the prototype TI-MBCT and (2) conducting a feasibility trial for an RCT for TI-MBCT for DVA survivors with PTSD.

#### Intervention refinement

The intervention refinement work is informed by the Medical Research Council (MRC) framework for the development and evaluation of complex interventions [31, 32] and prior research in the field of developing psychological interventions [33–35]. This study component will involve

1. An update of the 2014 literature review on adapting mindfulness programs for PTSD and DVA populations.
2. A qualitative study with women who have experienced DVA and professionals about using psychological interventions (including mindfulness-based approaches) for the treatment of PTSD in this population.
3. A consensus exercise with mindfulness experts on areas of uncertainty regarding the proposed adaptations to a standard MBCT program.

Findings from these three sources will be synthesized to produce the TI-MBCT prototype 2 intervention which will be tested and refined further in the subsequent feasibility trial.

#### Feasibility trial

**Fig. 1.**
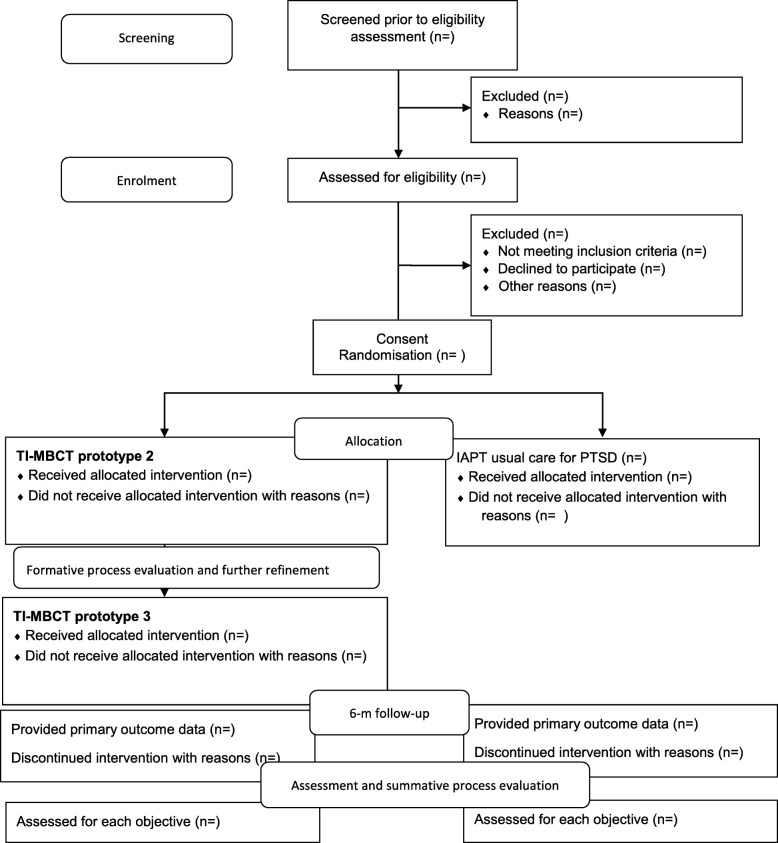
Flow diagram of randomized feasibility trial of two consecutive TI-MBCT groups vs NHS IAPT service https://www.spirit-statement.org/. TI-MBCT trauma-informed mindfulness-based cognitive therapy. NHS National Health Service. IAPT Improving Access to Psychological Therapies. PTSD Post-traumatic stress disorder

This study component comprises of a cycle of further refinement of the prototype TI-MBCT intervention through piloting and concurrent formative process evaluation. The direct experience of delivering and receiving the adapted TI-MBCT curriculum will feed into further intervention refinement in an iterative and systematic way. We will carry out a parallel-group, individually randomized, 2-arm feasibility trial with an embedded mixed-method process evaluation and economic evaluation (see Fig. 1).

From the pool of service users in third sector DVA agencies, women with PTSD will be recruited and randomized (2:1—intervention:control). The participants in the intervention arm will take part in a group-based TI-MBCT course and participants in the control arm will be asked to self-refer through the IAPT service. Participants will be followed up at 6 months post-randomization. In the intervention arm, we will run two consecutive TI-MBCT groups. The first group will receive the TI-MBCT prototype 2 produced through the intervention development phase. At the end of the first group, we will interview the participants to inform further intervention refinement and produce the TI-MBCT prototype 3 for the second intervention group. After the second group, we will invite participants to take part in further qualitative interviews, as part of a process evaluation. Results of the feasibility trial and process evaluation will inform a decision on progression to a full-size trial. If we find that it is feasible and acceptable to progress (see Table 1), we will produce a final version of the TI-MBCT manual for the full-size trial.

### Study setting

The study will be based at the University of Bristol.

#### Intervention refinement

Women who have experienced DVA and professionals will be recruited from non-NHS and NHS organizations, not limited by location. Interviews will take place at a location that is safe and convenient for both participant and researcher. The consensus exercise will be held on university premises.

#### Feasibility trial

Participants will be recruited from third sector DVA agencies in two local authorities in the southwest of England.

Study assessments and the TI-MBCT intervention will be delivered at locations that are safe and convenient for both participants and researchers, e.g., community centers. The control intervention will be delivered at NHS sites or non-NHS sites providing IAPT services for PTSD. Importantly, the intervention sites will be in geographical areas served by the participating third sector organizations from which the participants were recruited.

A safety protocol will be in place to protect the welfare of women who have experienced DVA participating in the trial. A safe method of contact will be agreed with the researchers, interviews will take place in a place safe for the researcher and participant, and under no circumstances will the researcher discuss any information with another person known to the participant.

### Eligibility criteria

The eligibility criteria for the *intervention refinement* process will be

Target population:

- Female
- 18 years or older
- Self-identified as victims or survivors of any forms of DVA
- Self-identified experience of mental health problem(s) including PTSD.

NHS-employed and non-NHS employed professionals:

- Mental health care practitioners: mindfulness practitioners and therapists with experience of treating PTSD.
- DVA advocates from third sector organizations with experience of working with people who have experienced DVA and PTSD.
- Managers of NHS-based and non-NHS based services for women who have experienced DVA.

The eligibility criteria for the *feasibility trial* will be

- Female
- 18 years or older
- Accessed collaborating third sector organizations providing health and social care to DVA victims and survivors
- Clinically important symptoms of PTSD [21] indicated by the baseline score above the clinical threshold on the Primary Care PTSD Screen (PC-PTSD-5). “Yes” to any exposure to traumatic events and “yes” to any three out of five items [36]

Women with and without children will be recruited to explore the feasibility of measuring mother-reported outcomes for children. If a woman has more than one child aged 8-18, the study investigator will use random number generation to select one child for the study to reduce the response burden on the woman.

The exclusion criteria for the feasibility trial are currently based on the standard MBCT guidance [37]; measures are summarized in Table 2:

- Unable to provide written informed consent
- Unable to speak and understand English (because interventions will be delivered in English)
- Current drug or alcohol dependency (the full Alcohol Use Disorders Identification Test (AUDIT) score ≥ 20, the Drug Use Disorders Identification Test (DUDIT) score ≥ 25)
- Organic brain damage
- Current or past psychosis
- Current persistent self-harm or suicide risk
- Already receiving psychological therapy

The inclusion/exclusion criteria for the feasibility trial will be revised and amended as necessary through the intervention refinement work.

**Table 2:** Schedule of measures in a feasibility trial

Procedures	Initial screening	Final screening	Baseline	6-month follow-up
Socio-demographics (bespoke)	x			
Speaking and understanding English (support worker’s judgment)	x			
Diagnosed psychosis, bipolar disorder, personality disorder (from collaborating agency case record)	x			
Current psychological therapy (from collaborating agency case record)	x			
Readiness to start mindfulness group or alternative talking therapy on the NHS (woman’s own judgment)	x			
The Primary Care PTSD Screen for DSM-5 (PC-PTSD-5) [36]. Cut off: “Yes” to any exposure to traumatic events and “yes” to any three out of five items.		x		
The Alcohol Use Disorders Identification Test Consumption (AUDIT-C), if The score is ≥ 5, full AUDIT [38, 39]. Cut-off ≥ 20.		x		
The Drug use Disorders Identification Test (DUDIT) [40, 41]. Cut-off ≥ 25.		x		
Depression and suicidal ideation as measured by the Patient Health Questionnaire-9 (PHQ-9) [42, 43]		x		x
Suicide history as measured by question “I made plans to end my life in the last 2 weeks” (bespoke)		x		
Suicide history as measured by question “I made attempts to end my life in the last 12 months” (bespoke)		x		
Support you have received for mental health problems (bespoke)			x	
The Life Events Checklist for DSM-5 (LEC-5) Standard [44].			x	
The PTSD Checklist for DSM-5 (PCL-5) [44]			x	x
The International Trauma Questionnaire (ITQ) [45]			x	x
The Severity of Dissociative Symptoms—Adult (Brief Dissociative Experiences Scale [DES-B]—Modified) [46]			x	x
Brief screening version of the Childhood Trauma Questionnaire [30]			x	
Composite Abuse Scale Revised-Short Version (events in the last 12 and 6 months, respectively) [47]			x	x
Self-Compassion Scale–Short Form (SCS-SF) [48]			x	x
Generalized Anxiety Disorder-7 (GAD-7) [49]			x	x
EQ-5D-5L [50]			x	x
KIDSCREEN-10 Index. Health Questionnaire for Children and Young People. Parent Version [51]			x	x
Resource questionnaire (bespoke)				x

### Sample size

#### Intervention refinement

The number of qualitative interviews conducted with women who have experienced DVA and professionals will be determined by the point at which thematic saturation is achieved, where further interviews are not expected to add additional information to the themes generated during the concurrent analysis. This is likely to include between 20 and 30 participants.

#### Feasibility trial

The required sample size is based on the precision for estimating the proportion (30%) of eligible participants consenting to participate in the study. If 120 eligible women are screened, then a true recruitment rate of 30% (= 36 recruits) will be estimated as between 21.8 and 38.2%, with 95% probability. This estimate is based on a previous trial that successfully recruited women from one of the collaborating DVA services [27, 52]. Based on previous trials of psychological interventions for women who have experienced DVA [27, 28] up to 33% of the consented and randomized participants are expected to drop out before the start of the mindfulness group/control intervention. Therefore, 54 will be randomized in order to be left with 36 who will be allocated to the intervention or control arm. We will compare the rate of drop out before starting mindfulness/control interventions between the two arms of the trial.

Retention will be optimized using established methods for trials involving women who have experienced DVA, [53–55] further modified through previous research in the UK context [56, 51], which resulted in 70–81% retention rates. The methods include maintaining a safe contact list, reimbursement for travel, childcare and £20 shopping voucher for each treatment session attended.

### Recruitment and consent

#### Intervention refinement

Women who have experienced DVA and professional stakeholders will be recruited through existing professional networks, previous study databases, and snowballing techniques. Participants will be sent the participant information sheet and consent form in advance, and written, or verbal, informed consent will be recorded.

#### Feasibility trial

Participants will be recruited through convenience sampling from a pool of service users in collaborating third sector organizations providing health and social care to DVA victims and survivors. The participants will be recruited in cohorts during a 2-month time-frames prior pre-scheduled TI-MBCT group dates:

- Near the time of exit from the service, support workers will approach women at individual sessions with study information and invitations
- Group work facilitators will approach women at the end of the group with study information and invitations
- After women exit the service, support workers will approach them during a 6-month follow-up phone call with study information and invitations
- Invitations will be sent to women from the waiting list of the agency services.

All service users undergo risk assessment within the service by their support workers. Support workers will carry out initial screening (Table 2). Those meeting the criteria and willing to be contacted by the study researcher will be referred to the study for final screening (Table 2). Participants will receive the participant information sheet and consent form in advance, and the study researcher will obtain informed written consent.

Randomization will be independent of the study researcher. Once informed consent has been secured and baseline data has been collected, the researcher will telephone a remote randomization service at the Bristol Randomised Trial Collaboration (BRTC) to ascertain the participant allocation: either to the intervention arm (mindfulness) or control arm (IAPT) in a 2:1 ratio, so that each participant has a 2/3rd’s probability of receiving the mindfulness intervention. The participant will be informed which arm she is assigned to by the researcher. Randomization will happen in blocks of 6. For every 18 participants that are randomized, we anticipate that six will drop out before the start of the intervention, four will continue in the study as controls and eight will continue in the study in the intervention arm—this will be sufficient to run a mindfulness group.

Allocation concealment will be achieved using the remote randomization service acting as a “third party” to assign the participant to either intervention or control arms. This ensures that the study researcher, enrolling and consenting participants, cannot be influenced by knowledge of their expected allocation at this stage. Once the participant has been allocated to either the intervention or the control arm, this assignment will be made available to the researcher. This is to enable the participant’s details to be passed onto the mindfulness teacher if they are in the intervention arm. The mindfulness teacher will invite the prospective intervention participants to an orientation meeting followed by an individual telephone assessment. Those participants randomized to the control arm will be advised by the study investigator how to self-refer to the IAPT service and helped with the self-referral if needed and wanted.

Due to the psychological nature of the intervention in both trial arms, it will not be possible to blind participants to details of which arm they have been allocated. Due to funding limitations, the same study researcher will carry out recruitment, baseline, and follow-up assessments, and monthly check-up calls; therefore, it will not be possible to blind the study investigator to the treatment groups. All process evaluation and analysis will be conducted by a different researcher blind to the participation group.

### Interventions

#### Intervention arm: TI-MBCT

The intervention will be group-based TI-MBCT developed through the intervention refinement phase of the study (see Table 3). The focus of this intervention is creating a new relationship with trauma-related phenomena and learning how to respond when the trauma of the past shows up in the present. TI-MBCT addresses the re-experiencing and reactivity characteristic of people who have experienced DVA with PTSD through the gradual development of skills for “de-centering” from distress. Instead of avoiding or becoming overwhelmed by distressing thoughts, powerful impulses, intense feelings, and disturbing body sensations, participants gradually learn to approach such experiences without judgment and with more acceptance and to gently ask “given that this experience is happening right now, how can I best take care of myself?” The cultivation of a non-judgemental attitude to what is arising in the present moment enables a way of responding, rather than reacting, to the self when trauma-related distress is experienced. In addition, being part of a group allows for a recognition that distress arising from past abuse is a shared experience, which reduces the sense of identification with being “damaged” that many people who have experienced DVA feel (Millband S.: How can an adapted MBCT course meet the specific vulnerabilities of women survivors of domestic violence and abuse? Unpublished thesis: Bangor University; 2015).

**Table 3:** TI-MBCT intervention overview

TIDieR* item	Description
Brief name	Trauma informed mindfulness-based cognitive therapy (TI-MBCT) [58, 59]
Why	TI-MBCT addresses the patterns of avoidance, re-experiencing, and reactivity characteristic of people who have experienced DVA with PTSD through the gradual development of skills for managing overwhelm and developing skills for “decentering” from distress
What	The manual, mindfulness practices and exercises for TI-MBCT will be developed through the intervention refinement process.
Who provided	An experienced mindfulness teacher with expertise in trauma, who is in supervision with a mindfulness-based supervisor with expertise in trauma.
How	TI-MBCT delivered in face-to-face groups of up to nine participants
Where	A site will be selected which is safe and convenient for both participants and the therapist (e.g., community centre).
When and how much	Once a week for eight weeks participants will attend a 2-h session and conduct 45 min of guided home practice.
Tailoring	The intervention will be refined to meet the needs of women with DVA trauma with a particular emphasis on establishing a sense of safety from which to turn towards challenging experiences.
Modifications	TI-MBCT will be refined during the study based on evidence synthesis from 1. A literature review on trauma-sensitive adaptations of mindfulness-based interventions 2. Qualitative interviews with women who have experienced DVA (including feasibility trial participants) and professional stakeholders 3. Consensus exercise with “experts by experience” of delivering mindfulness-based interventions to participants who have experienced trauma.
How well Planned Actual	Therapists’ records will be analysed to measure intervention uptake, retention and dose received. Home practice records completed by participants will be analysed to measure dose received. A standard tool for assessing fidelity of a mindfulness-based intervention [60] will be adapted to the TI-MBCT course and tested in the feasibility trial.

Note: *TI-MBCT* trauma-informed mindfulness-based cognitive therapy, *DVA* domestic violence and abuse

*Template for Intervention Description and Replication (TIDieR) checklist [61]

#### Control arm

Participants in the control group will be self-referred to the local IAPT service. Participants accessing the IAPT service receive an initial telephone assessment, after which they are offered different forms of therapy, based on the nature and severity of their symptoms and the availability of practitioners. At 6-month follow-up, we will collect IAPT data regarding each control participant’s individual care pathway to understand what psychological intervention(s) are provided as usual care.

### Outcomes and measures

The outcome of the *intervention refinement* component will be stakeholder views on the content and format for the TI-MBCT refinement obtained through semi-structured qualitative interviews.

#### Feasibility outcomes

Table 4 summarizes feasibility outcomes that will be used in the *feasibility trial* to evaluate the feasibility of a future full-size trial.

**Table 4:** Feasibility outcomes

Outcome	Description; measure
Recruitment/randomization rate	Proportion of participants randomized into two arms; the denominator will be the number of participants eligible for recruitment/randomization
Intervention uptake	Proportion of participants who took up the TI-MBCT or self-referred to the IAPT service; the denominator will be the number of participants randomized in the arm, respectively.
Intervention retention	Proportion of participants in the intervention arm who received the “minimum dose” of the intervention, four sessions of mindfulness intervention [29].
Follow-up rate	Proportion of participants followed up and providing outcome data at six months post-randomization out of those enrolled in the trial. The proportion who have been lost to follow-up will also be calculated by the trial arms.
Participant experience	Participant views on the acceptability of the intervention and trial design.

#### Intervention impact outcomes

Trial participants will complete standardized validated and bespoke questionnaires at baseline and 6-month face-to-face meetings with study researcher (Table 2). If we find that it is feasible and acceptable to collect the intervention impact data, these outcomes will be used in a full-size trial. The primary outcome will be clinically important symptoms of PTSD [21] captured by self-administered PTSD Checklist for DSM-V (PCL-5) [44] and International Trauma Questionnaire (ITQ) [45]. Secondary outcomes will include the severity of dissociative symptoms, depression, anxiety, DVA re-victimization, self-compassion, and woman reported child health and wellbeing.

#### Economic measures

A resource use data collection form will be developed for the trial, informed by that used in the Psychological Advocacy Towards Healing trial [56]. EQ-5D-5L will be used to collect data on participant health-related quality of life [50]. Responses will be converted to utility scores using the standard UK tariff values and this used to estimate quality-adjusted life years.

### Data collection methods

#### Intervention refinement

Qualitative data will be collected during semi-structured interviews with stakeholder professionals and women who have experienced DVA. Researchers will collect brief sociodemographic data (professional role/employment status, age, gender, ethnicity) at the outset, then use flexible topic guides to ensure primary issues are covered during each interview. Topic guides will be modified as necessary to address emerging findings and new lines of enquiry. A participant distress protocol will guide researchers during interviews about how to respond if participants become distressed during or after the interviews. Researchers will be supervised and have access to debriefing following participant interviews.

#### Feasibility trial

Data regarding feasibility will be obtained from study logs of recruitment and follow-up, therapists’ records about intervention uptake/sessions attended, retention and dose received, and interviews with participants about the acceptability of the intervention and trial design. Clinical data will be collected at face-to-face meetings with the researcher using the standardized validated questionnaires listed above. Economic data will be collated from the study logs detailing the cost of intervention delivery. Self-reported economic measures will be collected from participants 6 months post-randomization using paper-based, self-completed, and interviewer-assisted questionnaires.

### Data analysis

#### Qualitative analysis

Interview audio-recordings will be professionally transcribed and imported into NVivo software for line-by-line coding. The framework method [62] will be used to analyze the data in order to identify themes across the dataset, using constant comparison techniques. A second researcher will be involved in coding and reviewing themes. Data from the feasibility trial will first be analyzed as two separate data sets (intervention and control arms) to look at common themes within each arm, before making analytic comparisons across the data sets. Data will be explored for disconfirming cases within and across the arms of the study.

Data will be analyzed at two time points; initially after the first intervention group, in order to inform any further refinements of the prototype and subsequently, after the second intervention group to make comparisons across the two groups and time points, in terms of delivery and content of the intervention and the control group experience.

#### Quantitative analysis

Since this is a feasibility trial, the statistical analyses are mainly descriptive. We will calculate rates with 95% confidence intervals (CIs). Medians (ranges) will be calculated for ordinal data, means with standard deviations (SD) for continuous data, and raw counts (number, %) for nominal data. Differences in means (and 95% confidence intervals) between the two study arms will be presented for the various outcome scales, but no *p* values will be displayed. The SD of the outcome measures will be used to help inform a sample size calculation for a full trial.

#### Economic evaluation

The aim of the economic analysis is to inform the design of economic evaluation in the full-scale trial. The feasibility trial will be used to determine the optimal perspective, type of economic evaluation, scope, and data collection methods. The research team will design data collection tools, aiming to minimize missing data and identify relevant unit costs and the main cost drivers. The costs of the intervention and the control treatment will be crucial. An aim will be to establish how best to collect the data and how to value it.

Descriptive statistics will be reported for all quantitative and economic data collected. Missing data will be examined between baseline and follow-up interviews and prior to a full trial, with a view to establishing causes of missing data and how these might be addressed.

### Withdrawal criteria

Participants may withdraw because they no longer wish to attend the intervention or control therapy or assessment meetings or because a change in their health status or personal circumstances warrants their withdrawal. Researchers will attempt to contact participants up to three times using different modalities (e.g., text, email, and phone), before deeming them to be lost to follow-up. Data obtained up to the point of withdrawal will be anonymized and retained for analysis. Participants who have withdrawn or dropped out of the study will be contacted once by telephone and asked if they would agree to answer some questions to explore the reasons for their non-participation. This will be explained in the participant information sheet and consent form.

It is hoped that participants withdrawing from the intervention or control arm would remain willing to participate in follow-up measurements at 6 months; all participants withdrawing from the intervention/control phase will be approached once at the 6-month point, but contact will not be pursued if participants either (i) indicate that they do not wish to complete the follow-up stage or (ii) do not respond.

In the circumstance that a participant wishes to withdraw from the trial and therapy completely, they will no longer receive contact from the mindfulness teacher (if in the intervention arm), or from the IAPT therapist (if in the control arm) and will not be contacted about follow-up research appointments.

### Data management

Quantitative data will be entered in REDCap software. STATA will be used to conduct statistical analyses and produce graphs. Qualitative interviews will be audio-recorded on a University of Bristol approved encrypted digital recorder, transcribed, and analyzed in NVivo software. Information collected by the mindfulness teacher/IAPT therapist about the progression of the intervention will be recorded separately in the teacher/therapist logbook, and in pre-specified tables and worksheets.

We will follow the University of Bristol Data Protection Policy built on the General Data Protection Regulation. Personal data will be kept in a secure place—electronically in the study folder on the departmental file store with password-protected access and hard copies in a locked cabinet in a locked room on secure university premises. Personal data and an anonymization log will be kept separately from research data, and participants will be identifiable by study ID.

After the study has concluded, we will deposit the anonymized data in the University of Bristol Data Repository. Analyzed data will be retained for a minimum of 10 years.

### Study organization and management

The sponsor (University of Bristol) and funders (see below) had no role in the design of this study and will play no role in its conduct, data analysis, and interpretation, manuscript writing, or dissemination of results. The sponsor and funder will not control the final decision regarding any of these aspects of the study. The study management group (SMG) will assist the principal investigator in the day-to-day management of the project and meet monthly. A joint study steering committee and data monitoring and ethics committee (SSC/DMEC) of independent experts in DVA, PTSD, mindfulness, and statistics will act as an advisory group. The study is conducted in collaboration with a patient and public involvement (PPI) group consisting of women who have experienced DVA, who will advise on study materials and processes.

The study is conducted in accordance with the Research Governance Framework for Health and Social Care and Good Clinical Practice. The study will be monitored and audited in accordance with sponsor policies. All feasibility trial-related documents will be made available on request for monitoring and audit. Amendments to the protocol will be submitted to the Research Ethics Committee for approval.

### Adverse event reporting

All adverse events will be reported from the time the participant is enrolled in the feasibility trial until completion of the last study-related procedure. The research team will collect data on adverse events from study participants, researchers, and interventionists. Participants will be asked about adverse events at least every 30 days. If an adverse event were to occur, the study investigator or a delegated member of the study team would assess whether the event is serious or unexpected, and whether it is likely to be related to the study procedures or interventions. All adverse events will be recorded in the study file with a note that will identify when the event occurred, what happened, any potential study relation, action taken and resolution/closure of the adverse event. All serious adverse events will be reported to the University of Bristol research governance and ethics officer, who will notify the relevant Research Ethics Committee, the Insurance Office and the Research Governance Team as appropriate.

### Dissemination

The following are potential beneficiaries of the study: DVA survivors with PTSD, NHS and non-NHS mental health professionals, organizations providing DVA services, researchers in the area of trauma, mindfulness and DVA, commissioners of DVA, and mental health services.

Dissemination plan:

1. Study website with sections targeted at each beneficiary group.
2. At least two academic papers in peer-reviewed international journals.
3. At least two presentations at national and international conferences. Journals and conferences targeted at the multi-disciplinary audience will be prioritized. A national conference that is open to PPI members will be chosen, and one PPI member will be supported to attend.
4. Summary reports for professionals uploaded on the study website and sent to collaborating NHS networks, counseling services, general practices, and DVA agencies.
5. Plain English summary disseminated through an annual newsletter, the study website, websites of collaborating practices, and IAPT and DVA agencies.
6. Plain English summary sent to all study participants annually and at the end of the project.

## Discussion

DVA is common and destructive to health. PTSD is a major mental health consequence. Existing treatments for PTSD are often limited by high levels of discontinuation [23]. Women who have experienced DVA have specific needs because the trauma they have experienced is often recurrent and complex. NICE have called for research into psychological interventions for people who have experienced DVA [24]. Evidence is emerging that mindfulness-based therapies may benefit trauma symptoms [26]. However, research is needed to indicate whether TI-MBCT can be adapted to meet the needs of people who have experienced DVA with PTSD.

An evidence-based approach to intervention development and feasibility testing, based on co-production with stakeholders and iterative cycles of intervention refinement informed by process evaluation, may help to improve the acceptability of the intervention. Feasibility testing is important to this study, to address areas of uncertainty highlighted during intervention development. This includes the feasibility of recruiting DVA survivors to a group based psychological intervention and ways to optimize retention of this vulnerable population. The feasibility trial will allow researchers to assess the acceptability of randomizing participants and to clarify what treatment DVA survivors with PTSD are offered through the IAPT service.

DVA survivors with PTSD remain a group with unmet needs. The development of the TI-MBCT intervention and progression of the feasibility trial to full trial will contribute to the therapy evidence base for this population.

## Data Availability

Not applicable to the study protocol. After the study has concluded, the analyzed datasets will be available from NL on reasonable request.

## Abbreviations

DMEC: Data monitoring and ethics committee
DVA: Domestic violence and abuse
EMDR: Eye movement desensitisation and reprocessing
IAPT: Improving Access to Psychological Therapies
MBCT: Mindfulness-based cognitive therapy
NHS: National Health Service
NICE: National Institute for Health and Care Excellence
PPI: Patient and public involvement
PTSD: Post-traumatic stress disorder
RCT: Randomized controlled trial
SMG: Study management group
SSC: Study steering committee
TF-CBT: Trauma-focused cognitive behavioral therapy
TI-MBCT: Trauma-informed mindfulness-based cognitive therapy
